# Harnessing the Magic of the Dairy Matrix for Next-Level Health Solutions: A Summary of a Symposium Presented at Nutrition 2022

**DOI:** 10.1016/j.cdnut.2023.100105

**Published:** 2023-06-01

**Authors:** Allison L. Unger, Arne Astrup, Emma L. Feeney, Hannah D. Holscher, Dana E. Gerstein, Moises Torres-Gonzalez, Katie Brown

**Affiliations:** 1 National Dairy Council, Rosemont, IL, USA; 2 Department of Obesity and Nutrition Science, Novo Nordisk Foundation, DK-2900 Hellerup, Denmark; 3 Institute of Food and Health, University College Dublin, Dublin 4, Ireland; 4 Department of Food Science and Human Nutrition and Division of Nutritional Sciences, University of Illinois, Urbana, IL, USA

**Keywords:** bioaccesibility, cardiometabolic health, cardiovascular disease, cheese, food matrix, milk, nutrition guidance, precision nutrition, saturated fat, type 2 diabetes, yogurt

## Abstract

An emerging body of scientific evidence demonstrates that the food matrix—the interaction among nutrients, bioactive components, and physical structure of a food—can affect health in significant, unexpected ways beyond its individual nutrients. In particular, research suggests that consumption of dairy foods such as milk, yogurt, and cheese may affect human health in a matrix-dependent fashion. To disseminate and discuss the growing body of evidence surrounding the role of the dairy food matrix on cardiometabolic health, 3 expert researchers on the topic of the food matrix shared the latest science in a session entitled “Next-Level Health Solutions: The Magic of the Matrix” at the American Society for Nutrition’s 2022 LIVE ONLINE Conference. This article is a summary of the literature presented and discussed during that session. A substantial body of literature demonstrates that full-fat dairy foods, particularly fermented dairy foods, may beneficially modulate cardiometabolic outcomes depending on an individual’s health status. These findings have important implications for current authoritative dietary guidance that recommends the consumption of low-fat or fat-free dairy foods. Furthermore, this evidence may inform practical applications of harnessing dairy’s unique profile of bioactives for health promotion and disease prevention at the individual and community levels.

## Introduction

Modern nutrition science is rooted in efforts of the early 20th century that established the role of individual nutrients on health. This field of study has now evolved to recognize that the matrix of a whole food consumed, in addition to an individual’s characteristics and health status, also deserves consideration to fully capture the effect of diet on health. This paradigm shift in the field of nutrition has significant implications for current food-based nutrition guidelines that use a “one-size-fits-all” approach.

This article is a summary of a symposium entitled, “Next-Level Health Solutions: The Magic of the Matrix” at the American Society for Nutrition’s 2022 LIVE ONLINE Conference. During the symposium, 3 expert researchers on the topic of the food matrix particularly discussed the large body of evidence available that suggests that dairy foods, including full-fat and fermented varieties, influence cardiometabolic health in a manner dependent on the food matrix and an individual’s metabolic disease risk. Within this symposium, dairy foods were considered according to the United States Department of Agriculture (USDA) MyPlate definition (milk, yogurt, and cheese) with the exception of soy products [1]. Foods derived from milk that contain minimal calcium with a high-fat content, such as butter and cream, were not included as part of the dairy food group.

### Looking Beyond the Nutrition Facts Label: The Case for the Food Matrix

*The following is a summary of the first speaker session, presented by Dr. Hannah D Holscher* (Table 1).

**TABLE 1:** Session description of the symposium entitled “Next-Level Health Solutions: The Magic of the Matrix” presented at the American Society for Nutrition’s 2022 LIVE ONLINE Conference^1^.

Session title	Session subsections	Session objective	Session expert speaker
Looking beyond the Nutrition Facts Label: The case for the food matrix	1. The food matrix concept	Describe the evolution of nutrition science and the current understanding of the food matrix, including examples of the food matrix’s unexpected effect on nutrient bioavailability and health outcomes	Dr. Hannah D Holscher^2^
	2. Examples of the food matrix’s effect on nutrient bioaccesibility and health outcomes		
	3. Matrix-specific health effects and plausible biological mechanisms of the associated health effects		
			
Research supporting dairy matrix effects on health outcomes	1. Food versus nutrients: The matrix	Discuss emerging evidence on the diverse array of dairy-specific bioactive constituents and implications for human health	Dr. Emma L Feeney^3^
	2. Recent research findings on dairy foods		
	3. Dairy matrix effects and lipid profiles		
			
Applying the science: The role of the dairy matrix on precision nutrition	1. Saturated fat and cardiovascular disease	Evaluate the practical applications of harnessing the dairy’s unique profile of bioactives for health promotion and disease prevention with an emphasis on its place in the era of precision nutrition	Dr. Arne Astrup^5^
	2. The role of the dairy matrix and cardiometabolic disease^4^		
	3. One diet does not fit all: A role for precision medicine		

1 Sessions are listed in a sequential order. Each session was approximately 15 min in length as part of a 60-min symposium, followed by 15 min designated for question and answer by the audience.

2 Department of Food Science and Human Nutrition and Division of Nutritional Sciences, University of Illinois, Urbana, IL, USA.

3 Institute of Food and Health, University College Dublin, Dublin 4, Ireland

4 The complete subsection title presented at Nutrition 2022 was “The role of the dairy matrix and cardiometabolic disease and bone health.” As the scope of the symposium was predominantly focused on evidence related to dairy consumption and cardiometabolic health outcomes, the revised subsection title reflects the omission of the limited discussion of bone health outcomes within this synopsis.

5 Department of Obesity and Nutrition Science, Novo Nordisk Foundation, DK-2900 Hellerup, Denmark.

Lifestyle patterns, particularly diet composition, profoundly affect health spanning cardiometabolic health, bone health, adiposity, and neurocognition [1]. Historically, advances in the understanding of the relationship between diet and health have been propelled through a reductionist lens, focusing on the biological responses attributed to individual nutrients consumed [2,3]. However, researchers are increasingly shifting focus from this nutrient-driven approach and contributing to a growing body of evidence that indicates that the health effects of specific nutrients are influenced by their vehicle of consumption, i.e., the “food matrix effect” [4,5].

The food matrix is defined by the USDA as the “nutrient and non-nutrient components of foods and their molecular relations.” The role of the food matrix in the interaction between a food’s nutrients and health outcomes is believed to occur, partly, because of differences in the bioaccesibility of nutrients within whole foods [4,6]. Research supports that the physical structure of a food significantly influences the fraction of a compound (e.g., nutrient or bioactive) liberated, and thus digested and absorbed, from its matrix in the gastrointestinal tract when consumed [4,7].

### Examples of the food matrix’s effect on nutrient bioaccesibility and health outcomes

When considering the anatomy of plant-based foods, plant matrices contain encapsulated starch granules and different types of fibers within the cell wall [6]. Plant fibers are typically resistant to digestion; however, external factors such as temperature, pH, processing, and fermentation will alter the accessibility of these plant-based nutrients [6]. For example, the grinding of almonds (i.e., as a paste) disrupts the plant’s cell walls, thereby increasing its metabolizable energy content compared with that of their whole counterparts [8]. Observed changes to the physical structure of various foods by other external factors lend support to this concept as well. Heating and cooling of pasta can render starch resistant to digestion, affecting the glycemic response to consumption [6,9]. Furthermore, cooking eggs actually enhances the bioavailability of biotin [10].

An additional consideration in the influence of food processing techniques on the bioaccesibility of nutrients early in the gastrointestinal tract is the consequential effect on substrate availability for the intestinal microbiota [[11], [12], [13]]. This cumulative effect of matrix-specific metabolism of food components within the gastrointestinal tract and by the intestinal microbiota has implications for physiologic and personalized responses to foods and their nutrients and, thus, disease risk [14,15]. Indeed, a growing body of in vitro, preclinical, and clinical research compellingly indicates that nutrients consumed within a food matrix compared with those in isolation can have a meaningful impact on an individual’s health status, such as blood glucose and blood lipid profiles [4,14]. In summary, science supports the merit of future public health efforts to look beyond the Nutrition Facts label and be inclusive of the current science demonstrating the powerful connection among nutrients, the food matrix, and health.

### Matrix-specific health effects and plausible biological mechanisms of the associated health effects

#### Case study: Almonds

Nuts are nutrient-dense foods that contain fiber, unsaturated fatty acids, and polyphenols, along with a rich content of other fats, protein, and micronutrients [16,17]. Epidemiologic and intervention trials have demonstrated that nut consumption can promote health and well-being by positively modulating cardiovascular health, blood glucose control, and body composition [16,17].

Gebauer et al. [8] performed a crossover clinical trial in healthy American adults to determine how various forms of food processing affected nutrient bioaccesibility of almonds. The intervention was designed as a 3-wk treatment period with a 1-wk washout between each of the following 5 treatments: *1*) base control diet alone or base diet supplemented with *2*) whole natural almonds, *3*) whole roasted almonds, *4*) chopped, roasted almonds, or *5*) almond butter. An analysis of the different almond forms showed that processing affected the physical properties of almonds. Notably, whole natural almonds had a greater hardness with a lower number of particles per image (i.e., broken into fewer, larger pieces) and lower metabolizable energy than other almond forms. In follow-up analyses, these differences in metabolizable energy were found to coincide with changes in intestinal bacteria [18]. These results shed light on the potential mechanisms underlying observed beneficial health effects after nut consumption. More broadly, these results provide evidence that the processing of a food can mediate the relationship between nutrient consumption and health.

#### Case study: Dairy

**FIGURE 1:**
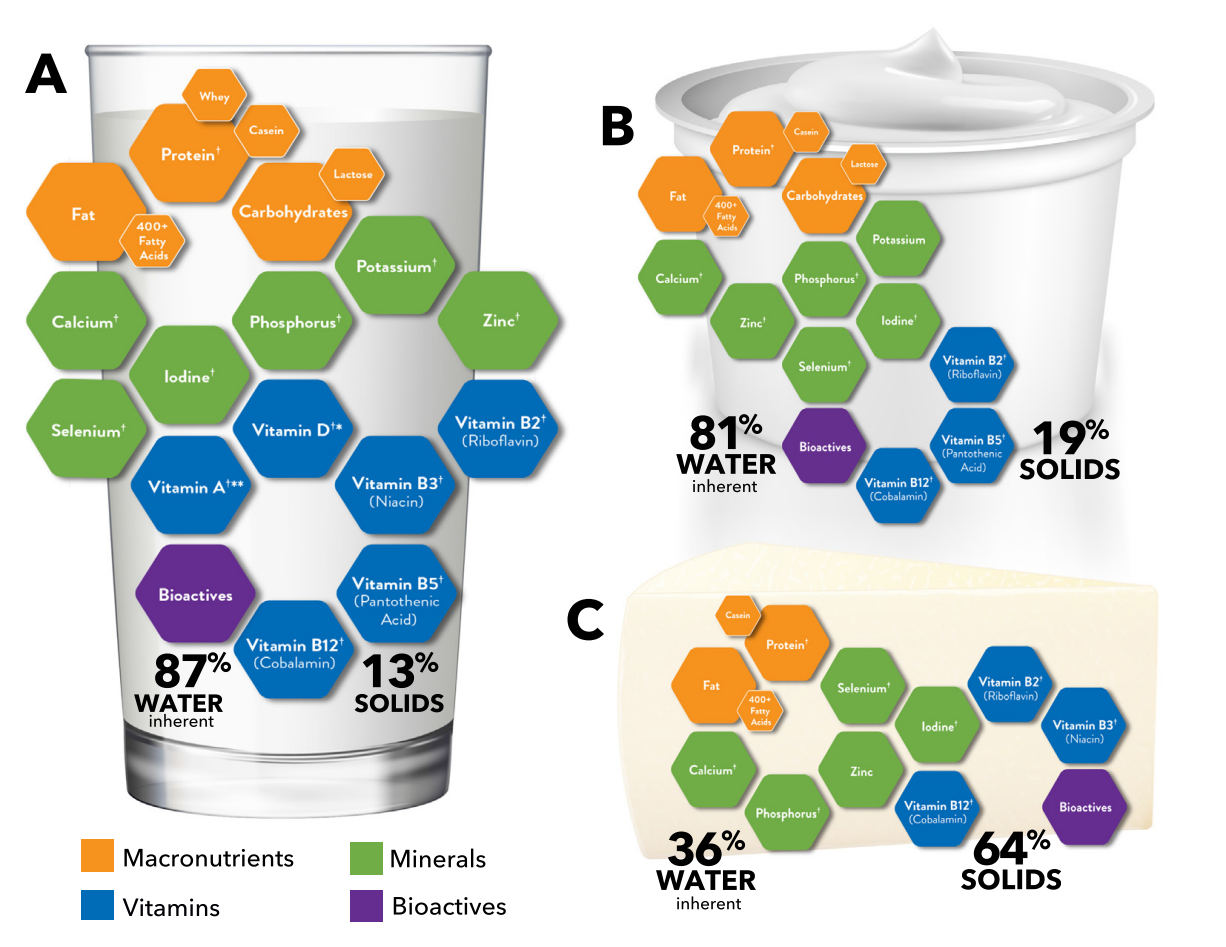
A graphical representation of the dairy food matrix of milk (A), yogurt (B), and cheese (C). (A) The graphic is based on the average composition of whole (3.25%) milk (FDC ID: 746782 Foundation). ^†^Milk is a good or excellent source of 13 essential nutrients. ∗Vitamin D is added to milk. ∗∗Vitamin A is naturally occurring in whole milk and added to reduced-fat, low-fat, and fat-free milks. (B) The graphic is based on the average composition of low-fat (1%) vanilla yogurt (FDC ID: 170888 SR Legacy). ^†^Yogurt is a good or excellent source of 9 essential nutrients. (C) The graphic is based on the average composition of cheddar (29% fat) cheese (FDC ID: 328637 Foundation). ^†^Cheese is a good or excellent source of 8 essential nutrients. The following are criteria for inclusion in the graphics (A–C): *1*) The nutrient contributes ≥10% of the DV per reference amount customarily consumed (RACC) based on data from USDA’s FoodData Central (or another government resource if unavailable in the USDA database); *2*) it is a nutrient of public health concern for the United States population: vitamin D, calcium, potassium, or fiber AND it contributes ≥8% of the DV per RACC; and *3*) macronutrients are included if they contribute ≥2% of the DV per RACC based on data from USDA’s FoodData Central. Graphics adapted from the National Dairy Council [20].

Similar to plants, the food matrix effect on bioaccesibility is apparent across different types of dairy foods and reflective of distinct processing methods [19]. Dairy foods (milk, yogurt, and cheese) comprise a food group hallmarked as a nutrient-rich contributor of essential nutrients to the diet, such as high-quality protein, calcium, vitamin D, potassium, and several other vitamins and minerals (Figure 1) [5,20]. In addition, dairy foods are important sources of bioactives, defined as “food constituents other than those that meet basic nutritional needs that are responsible for a change in human health.” Dairy components gaining recognition as bioactives include dairy-derived fatty acids, phospholipids, peptides, and the milk fat globule membrane (MFGM) [19,[21], [22], [23]]. Although milk with its liquid matrix is the primary ingredient of all other dairy foods, the nutrient and bioactive composition and physical structure of milk shift markedly during processing to yogurt (gel matrix) and cheese (semisolid or solid matrix) [5].

Research has demonstrated that the bioaccesibility and physiologic response of specific dairy-derived nutrients may be affected by its food matrix (Table 2) [[24], [25], [26], [27], [28], [29], [30], [31], [32], [33], [34], [35]]. In a small, randomized crossover study conducted by Bendsen et al. [32], fecal fat excretion was observed to be higher in participants who consumed high-calcium dairy foods than that in those who consumed low-calcium dairy foods (2300 and 700 mg calcium/d, respectively). Soerensen et al. [24] also observed that calcium consumption from a diet containing milk or cheese enhanced fecal fat excretion in healthy, young adult men compared with an isocaloric control diet. No differences in fecal fat excretion were found between diets containing milk or cheese; however, a correlation was observed between fecal fat excretion and diet-induced changes in LDL cholesterol concentration among participants. Taken together, these studies support a large body of evidence that the dairy food matrix exerts a beneficial effect on health, particularly body composition and cardiometabolic health [15,35].

**TABLE 2:** A summary of scientific evidence describing the beneficial properties of the dairy matrix on cardiometabolic disease risk factors and health outcomes as presented and discussed at the symposium entitled “Next-Level Health Solutions: The Magic of the Matrix” at the American Society for Nutrition’s 2022 LIVE ONLINE Conference^1^.

	Risk factor				Health outcome			
	Blood glucose control	Body weight or composition	Blood lipids	Blood pressure	CVD	Type 2 diabetes	Gastrointestinal health	Mortality
Dairy food								
Milk	—2	✓^3^ [24]	✓ [24]	—	—	—	—	—
Yogurt	—	—	—	—	✓ [25]^4^	✓ [26,27]^4^	—	✓ [25]^4^
Cheese	—	✓ [24]	✓ [24,28–30]	—	✓ [25,31]	✓ [27]^4^	—	✓ [25]^4^
Dairy-derived nutrient								
Calcium	—	✓ [24,32]	✓ [24]	—	—	—	—	—
Fat/fatty acids	✓ [33]	✓ [34]^5^	—	—	—	✓ [33]	—	—
Dairy-derived bioactive								
Exopolysaccharides	—	—	—	—	—	—	✓ [15]	—
Peptides	—	—	—	✓ [15,33]	—	—	—	—
Probiotics	—	—	—	—	—	—	✓ [15,35]	—

1 Research displayed includes primary and review articles that were presented and discussed by the speakers during the symposium session.

2 Dash symbols indicate that research was either unavailable or not discussed by the speakers during the symposium session.

3 Checkmark symbols represent results from evidence presented and discussed by the speakers during the symposium session.

4 Reference cited displays results for cheese and yogurt consumption when grouped as total fermented dairy consumption.

5 Reference cited does not specifically evaluate dairy fat.

Much recent scientific interest has focused on the unique health properties of fermented dairy foods such as yogurt. Yogurt is created through microbial fermentation of milk by the lactic acid bacteria *Streptococcus thermophilus* and *Lactobacillus bulgaricus*, resulting in its gel-like structure and contributing to the presence of bioactives and health-promoting properties [36]. For example, peptides that form during dairy fermentation may exert antihypertensive activities [15,33]. Other bioactives such as exopolysaccharides, produced by lactic acid bacteria, play a structural role in yogurt by increasing its viscosity and possibly contribute to gastrointestinal immunity [15,37]. In addition, β-galactosidase, derived from the live microorganisms in yogurt, contribute to lactose digestion within the intestines, which can provide a notable benefit to lactose maldigesters [38]. The interaction between probiotics and its dairy food matrix (most commonly yogurt and kefir) may beneficially modulate the symptoms of gastrointestinal distress such as diarrhea and constipation in a strain-dependent fashion [35]. In vitro research has shown that the unique viscosity of yogurt enhances the viability of probiotics by protecting it during simulated oral, gastric, and intestinal digestion [39,40]. Other mechanisms of improved probiotic survivability when consumed as part of yogurt have been proposed, such as the contributions of the yogurt to buffering stomach acidity, protection within the gastrointestinal tract through lactic acid bacteria–derived exopolysaccharides or MFGM, and the presence of lactose as an energy source for the probiotics [39,40].

### Research Supporting Dairy Matrix Effects on Health Outcomes

*The following is a summary of the second speaker session, presented by Dr. Emma L Feeney* (Table 1).

### Food versus nutrients: The matrix

In nutrition research, there are several different methods to assess the effect of diet on health [2]. When investigating the role of a diet on a particular health outcome, scientists may choose to focus on food constituents (e.g., dietary fiber), food items (e.g., apples), or food groups (e.g., fruit) [2]. The classic reductionist framing to nutrition science has focused on examining food constituents. Importantly, evidence resulting from this approach has led to significant advancements in the understanding of the health properties of individual nutrients. One prominent example of these scientific efforts is calcium’s well-known essential contribution to optimal bone health. However, an important shortcoming of reductionism when applied to nutrition science is its inability to holistically assess the influence of overall meals and dietary patterns on health. Indeed, a review by Aguilera et al. [4] discusses the importance of considering the interaction of a food’s nutrients, matrix, and microstructure on subsequent digestion and absorption dynamics.

### Recent research findings on dairy foods

This increased recognition of the food matrix to influence the relationship between nutrient intake and health outcomes has significant implications for research examining dairy’s role on cardiometabolic health. In 2021, a meta-analysis by Jakobsen et al. [31] reported that high-fat milk consumption was associated with CHD risk (8% increased risk per 200 g increased consumption per day) (Table 2). Total and low-fat milk consumption was not associated with CHD risk. However, in the same meta-analysis, cheese consumption was associated with a reduced risk of CHD (4% reduced risk per 20-g increased consumption per day). This result is notable because cheese, on an average (25% fat), is a dairy food with a higher content of total fat, saturated fat, and sodium than milk [5]. However, cheese also typically has a higher content of several vitamins and minerals (e.g., calcium and phosphorus), MFGM, and protein [5]. In addition, cheese as a fermented dairy food has a distinct physical structure, protein network and composition, and MFGM structure compared with milk (reviewed in detail in the study by Thorning et al. [5]). Thus, it is important to consider the role of the food matrix when examining the health effects of dairy as a food group.

### Dairy matrix effects and lipid profiles

Findings published by Feeney et al. [28] underscore the potent protective effect of dairy fat on blood lipids concentrations when packaged in a fermented dairy food matrix such as cheese [28,29]. In a parallel intervention trial, healthy overweight adults (aged 50 y or older, *n* = 164) were randomly assigned to consume ∼40 g of dairy fat for 6 wk through 1 of the following 4 treatments: *1*) full-fat cheddar cheese (group A), *2*) reduced-fat cheddar cheese and butter (group B), *3*) butter (group C), or *4*) full-fat cheddar cheese per group A but with an additional 6-wk run-in period that excluded cheese consumption (group D) [28]. After the intervention, a stepwise reduction in total cholesterol (TC) and LDL cholesterol was observed in a matrix-dependent fashion. In particular, the highest reduction in TC and LDL cholesterol was observed when participants consumed all dairy fat packaged within the cheese matrix (group A), whereas the least reduction was observed in participants who consumed all dairy fat in the form of butter (group C). A subsequent exploratory analysis suggested the observed reductions in LDL cholesterol were driven by reductions in overall LDL particle concentration and small (i.e., potentially more atherosclerotic [41]) LDL particles in particular. However, LDL particle sizes were not affected by the dairy food matrix and participants across all diet groups were found to have similar particle size distributions [30]. A separate secondary analysis ranked all participants, regardless of assigned diet, into tertiles for each lipid response after the study intervention [29]. In particular, those with the highest reduction in TC or LDL cholesterol concentration, tertile 1, were designated as responders, whereas those with the highest increase in TC or LDL cholesterol concentration, tertile 3, were designated as nonresponders (*n* = 34–35 per tertile) [29]. When examining changes in TC and LDL cholesterol concentrations in groups A–C, a higher percentage of responders and nonresponders clustered in the cheese (A) and butter (C) diets, respectively [29]. Cumulatively, this body of evidence substantiates that health implications of cheese, and perhaps other fermented dairy foods, are “more than the sum of their parts,” particularly, regarding saturated fat content.

Data from the intervention trial conducted by Feeney et al. [28] also underlines the importance of considering interindividual variation in physiologic responses to dairy fat and its matrix [29]. Notably, when again considering groups A–C only, participants demonstrating the largest percentage decrease (i.e., responders) in TC concentration showed significantly higher concentrations of TC and HDL cholesterol concentrations and lower concentrations of triglycerides than nonresponders at baseline. Similar findings were observed in those with the largest percentage decrease in LDL cholesterol. In addition, a multiple regression analysis revealed that the changes in TC and LDL cholesterol concentrations were associated with baseline TC concentration, triglyceride concentration, body weight, and high-sensitivity C-reactive protein concentration. Thus, baseline blood lipid concentrations may be a useful predictor of the efficacy of personalized dietary intervention strategies to improve cardiometabolic health and reduce disease risk.

Collectively, results from this clinical trial contribute to the growing evidence that certain foods may optimally promote health on a personalized basis depending on an individual’s characteristics and health status. However, more research is needed to understand how different food matrices within meals and overall dietary patterns affect health and disease risk across the lifespan. For example, in vitro research indicates that distinct matrices among cheese varieties (e.g., cottage cheese compared with parmesan) alter digestion and absorption behavior [5,19]. Follow-up research should also consider the differences in food processing and preparation methods before consumption (heating and cooling) on digestion, absorption, and its effects on health.

### Applying the Science: The Role of the Dairy Matrix on Precision Nutrition

*The following is a summary of the third speaker session, presented by Dr. Arne Astrup* (Table 1).

### Saturated fat and CVD

The diet–lipid–CVD hypothesis (also referred to as the lipid hypothesis or diet–heart hypothesis) was established and incorporated into dietary recommendations over 50 y ago. This concept posits that consumption of saturated fat leads to high concentrations of blood cholesterol, atherosclerosis, and, ultimately, CVD [42]. Current dietary guidance in the United States and Europe continues to lean on this rationale to maintain advocacy for the restriction of saturated fat in the diet [1,43]. Simultaneously, a growing body of evidence published largely over the past 2 decades has failed to convincedly demonstrate a strong causal relationship between consumption of saturated fat, as a nutrient class, and poor heart health. In 2020, a Cochrane analysis by Hooper et al. [44] reviewed 15 randomized controlled trials (all designed for a minimum of 24 mo duration; 56,675 total participants) assessing the effect of saturated fat consumption on CVD end points. These authors reported no effect of reducing saturated fat consumption on CHD-related, CVD-related, or total mortality; fatal or nonfatal heart attacks; or CHD events or strokes. Of note, an initial finding by the authors of decreased risk of CVD events after reduction of saturated fat consumption was attenuated in a sensitivity analysis that excluded studies that aimed, but failed, to reduce saturated fat consumption among participants.

The relevance of the diet–lipid–CVD hypothesis has been further scrutinized with the concurrent emergence of literature that reveals that blood LDL cholesterol concentrations may not be the most appropriate biomarker to determine an individual’s CVD risk [45]. LDL cholesterol comprises subclasses of particles with differing cholesterol content, and updated evidence highlights that these distinct particle subclasses carry divergent atherogenic properties [41]. Large LDL particles are primarily observed to act benignly on cardiovascular health [46]. By contrast, small dense LDL particles are more strongly associated with the development of atherosclerosis [46]. Furthermore, evidence shows that large LDL particle concentrations are primarily the LDL subclass influenced by dietary saturated fat, whereas small dense LDL concentrations are dictated more so by carbohydrate intake [46].

Dairy foods exemplify the complexity and nuance surrounding the relationship between saturated fat consumption and health. Dairy fat is uniquely diverse, with ∼60% being saturated fat but comprising >400 distinct types of fatty acids (Figure 1) [47,48]. Within this vast assortment of fatty acids there are short-chain, medium-chain, odd-chain, and branched-chain fatty acids—all of which are structurally defined as saturated fats but differ in their metabolic fates and health properties [22,49]. Another distinguishing feature of dairy fat is the MFGM, a lipid trilayer housing most of the dairy fat, which is rich in bioactive sphingolipids, phospholipids, and peptides [5,50]. Despite purported expectations from consumption of a food rich in saturated fat, evidence supports that the distinct fatty acid profile of dairy as part of its complex food matrix contributes to dairy’s protective effects on cardiometabolic health outcomes (reviewed in detail by Mozaffarian and Wu [33]). Taken together, it is increasingly clear that the diet–lipid–CVD hypothesis and the stigma surrounding saturated fats as a single group of nutrients to be restricted or eliminated from the diet are overly simplistic and warrant reconsideration within global food-based health recommendations [51,52].

### The role of the dairy matrix and cardiometabolic disease

A large body of evidence supports that dairy consumption may exert protective effects for health through a complex network of complementary and synergistic mechanistic pathways (Table 2) [33]. In particular, research assessing the role of fermented dairy consumption on cardiometabolic health provides compelling evidence for the concept of the food matrix effect in dairy foods. For example, in 2017, Guo et al. [25] reported findings from a dose–response meta-analysis showing a neutral relationship between total (high-fat or low-fat) dairy or milk consumption and outcomes of mortality and CVD. However, this analysis also found a modest inverse association for total fermented dairy (such as yogurt and cheese) consumption and these same end points. This protective relationship between cheese consumption and cardiometabolic health supports similar conclusions of studies and meta-analyses published in the past decade [26,27]. Several components within dairy’s unique food matrix may be pivotal to these beneficial biological responses, leading to reduction of cardiometabolic disease risk, such as calcium and the diverse composition and structure of dairy fat [33]. In addition, several unique bioactives have been noted to arise from the fermentation process of yogurt and cheese, such as short-chain fatty acids and peptides produced by bacteria, which may contribute to insulin sensitivity and reduced blood pressure, respectively [15,33].

In 2012, a meta-analysis of randomized controlled trials revealed that inclusion of dairy in the diet affects body composition favorably (i.e., decreased fat mass and increased lean mass) [53]. Similarly, a meta-analysis of randomized controlled trials by Chen et al. [54] revealed that dairy consumption in an energy-restricted diet facilitated body weight, and particularly body fat, reduction. This protective effect of dairy consumption on cardiometabolic health continues to be shown in more recent research. An overview of systematic reviews and meta-analyses in 2019 reported an observed dose–response inverse relationship between dairy product consumption and type 2 diabetes (T2D) risk [26].

### One diet does not fit all: A role for precision medicine

Scientific awareness has evolved to recognize the complex interplay of interindividual variation in physiologic responses to diet and disease progression. Thus, it is important to consider that a personalized approach may be more appropriate in many instances, as opposed to a one-size-fits-all approach, to reflect advancements in the understanding of nutrition and health over the past 50 y.

Specific to dairy, such generalized nutrition guidance is evident in global recommendations for all adults to consume low-fat or fat-free dairy foods [55,56]. However, this guidance disregards the mounting evidence that full-fat dairy foods are not associated with cardiometabolic disorders, such as insulin resistance, hypertension, CVD, and T2D [33,51]. Furthermore, research supports that full-fat dairy may reduce cardiometabolic disease occurrence, particularly in at-risk individuals [34,51]. For example, a meta-analysis published in 2000 (16 trials; 1910 total participants) highlighted that an ad libitum low-fat, high-carbohydrate diet can lead to body weight loss or maintenance [57]. However, more recent research suggests that a carbohydrate-restricted diet may be more effective for weight reduction in individuals with T2D [58]. In 2017, Astrup et al. [34] discussed a body of evidence indicating that normoglycemic participants lost more weight on a low-fat diet, whereas participants with T2D were more responsive to a low-carbohydrate diet. A leading explanation for differential physiologic responses to high-fat/low-carbohydrate compared with low-fat/high-carbohydrate diets is the role of satiety signaling. Carbohydrate content in the brain is critical to feelings of satiety, and there is a growing scientific understanding that insulin resistance, a key trait of prediabetes and T2D, can interfere with glucose uptake in the brain and, thus, diminish normal satiety signaling [59,60]. Importantly, most of the population worldwide is now insulin resistant to some degree. In the United States, ∼40% of the population have prediabetes, with another 10%–15% of the population having T2D [61].

To summarize, a large body of research over the past 2 decades supports that there is insufficient evidence to warrant avoiding consumption of saturated fat as a strategy to reduce body weight or cardiometabolic disease risk. Furthermore, full-fat dairy foods, particularly fermented foods such as yogurt and cheese, may optimally protect against T2D and CVD risk among insulin resistant, at-risk individuals. Modern nutrition science has demonstrated that the dairy food matrix comprises a complex network of nutrients and bioactives that contain health-promoting properties. Nevertheless, more studies are needed to fully understand the personalized effect of foods and dietary patterns on health. Moving forward, the preponderance of evidence suggests that consideration of a food’s matrix rather than only its nutrient profile is warranted for adoption into food-based nutrition guidance.

## Conclusion

Since its inception in the 1970s, federal nutrition guidance has used a broad, one-size-fits-all approach for dietary recommendations to the general public [62,63]. This strategy to date has been fundamental to establishing nutrition standards for community-level and population-level guidelines and has been pivotal for progress to advance health and wellness globally. One notable example is the critical role that nutrition guidance plays for federal feeding programs to promote nutrition security and health in school-aged children (i.e., USDA National School Meals) [64]. Importantly, continued application of generalized language in nutrition guidance is likely to remain an appropriate and sufficient approach for certain instances or for some populations, such as healthy children. However, the rising prevalence of chronic diseases globally points to an urgent need for updated nutrition guidance that recognizes the role of personalized food and dietary pattern choices to optimally promote health and well-being at the individual and community levels.

Overall, consumption of dairy foods is associated with protective effects on cardiometabolic health. Furthermore, a growing body of scientific literature demonstrates that full-fat and fermented varieties of dairy foods may be superior for health promotion depending on an individual’s disease risk. Sustained research and public health efforts are needed that prioritize gaining a better understanding of matrix-dependent health properties of dairy foods and how dairy foods within overall eating patterns can be harnessed based on an individual’s characteristics and needs. Such initiatives will be paramount to advancing effective guidance to reduce the burden of diet-related disease and improve public health worldwide.

## Funding

This article is intended as a synopsis of presentations made by AA, ELF and HDH at the American Society for Nutrition 2022 LIVE ONLINE Conference 14–16 June 2022. The ASN Nutrition 2022 session that this article is based on was supported by National Dairy Council. This support included honoraria for ELF and HDH.

The authors reported no funding received for this study.

## Author disclosures

ALU, DEG, MT-G, and KB were the employees of the National Dairy Council (NDC) at the time this article was written. AA reports no conflict of interests related to the current topic. AA is a member of scientific advisory boards of Groupe Éthique et Santé (France), International Egg Nutrition Centre (United Kingdom), and Green Leaf Medical, Sweden; a member of the International Carbohydrate Quality Consortium; an associate editor of the *American Journal of Clinical Nutrition*; and a co-owner of various patents owned by the University of Copenhagen. ELF has received speaking honoraria from the NDC and the EU milk forum. HDH has received speaking honoraria from NDC.
